# Impact of Population Stratification on Family-Based Association in an Admixed Population

**DOI:** 10.1155/2015/501617

**Published:** 2015-05-04

**Authors:** T. B. Mersha, L. Ding, H. He, E. S. Alexander, X. Zhang, B. G. Kurowski, V. Pilipenko, L. Kottyan, L. J. Martin, D. W. Fardo

**Affiliations:** ^1^Divisions of Asthma Research, Biostatistics and Epidemiology, Human Genetics, and Physical Medicine and Rehabilitation and Center for Autoimmune Genomics and Etiology, Cincinnati Children's Hospital Medical Center, Cincinnati, OH 45229, USA; ^2^Department of Pediatrics, University of Cincinnati College of Medicine, Cincinnati, OH 45267, USA; ^3^Health Services Administration, Xavier University, Cincinnati, OH 45207, USA; ^4^Department of Biostatistics, University of Kentucky, Lexington, KY 40536, USA

## Abstract

Population substructure is a well-known confounder in population-based case-control genetic studies, but its impact in family-based studies is unclear. We performed population substructure analysis using extended families of admixed population to evaluate power and Type I error in an association study framework. Our analysis shows that power was improved by 1.5% after principal components adjustment. Type I error was also reduced by 2.2% after adjusting for family substratification. The presence of population substructure was underscored by discriminant analysis, in which over 92% of individuals were correctly assigned to their actual family using only 100 principal components. This study demonstrates the importance of adjusting for population substructure in family-based studies of admixed populations.

## 1. Introduction

Complex diseases result from the interplay of multiple genetic and environmental factors. To study the genetic basis of complex diseases, two broad types of study designs—population-based and family-based—are often used. Population-based designs sample individuals who are unrelated, as in case-control studies. Family-based designs use related individuals, often sampled through a proband. Case-control design is based on allele or genotype frequencies comparison of unrelated affected and unaffected individuals in the population [1]. An allele in a gene is said to be associated with a trait if it occurs at a significantly different frequency in the affected individuals compared to the control group (i.e., when the null hypothesis of equal allele frequency across groups is false). Family-based designs use groups of trios, nuclear families, or extended families. Family studies can address whether a disease aggregates in families [2]. Such studies typically examine correlations between traits and deviations from the allele transmissions expected assuming Mendelian inheritance. Although case-control designs have practical advantages over family-based designs in sample recruitment and collecting DNA from unrelated cases and controls, family-based association studies have received much attention in the literature because of their robustness to population stratification and higher power to detect very rare variants with small effects compared to case-control studies. Population stratification is present when the population includes several subpopulations, and the allele frequency of interest differs in each subpopulation due to systematic differences in ancestry rather than association of variants with disease [3, 4]. Although most population stratifications occur when there are multiple races or ethnicities in case-control study design, significant population stratification can be identified even within an apparently homogeneous North American population of European ancestry [5]. Recent study found that individuals, who are identified as white, have about 3.5% non-European ancestry [6].

By contrast, family-based association analyses assume that individual family members come from a common genetic background, and families tend to be more homogeneous regarding exposure to environmental factors that may be associated with the disease etiology. Hence, the analysis of phenotypes among family members is moderately controlled for both genetic background and environmental exposures. Because family members share a predictable proportion of their genes identical by descent, the background genetic variation is somewhat controlled as a function of the degree of relationship (or kinship coefficient) and modeled as a polygenic component [7]. This strong assumption of population homogeneity, however, is often untenable, and many association studies include samples from structured family members or admixed individuals. The world is becoming highly multiethnic, and intermarriage between different groups is becoming more and more common [8]. In the United States, the two major admixed populations are the African and Latino Americans. Populations like African Americans and Latino Americans were formed within the past 400 years (i.e., within approximately 10 generations) [9]. Therefore, the standard approach of selecting all individuals from the same population/ethnic group and geographic area is not always possible. In an extended family, many of the loci may be unique or may greatly vary in frequency within and between family members. False-positive associations (Type I errors) occur when the frequencies of genetic markers and the disease of interest vary across different subpopulation groups [10, 11]. Data from the San Antonio Family Study (SAFS) provided through Genetic Analysis Workshop 18 [12] are a classic example in which related individuals were recruited from admixed Mexican American families. In such situations, failure to appropriately account for pedigree structure in family-based study can lead to spurious associations. Here, we consider accounting for family structure in admixed ancestry. Using an admixed population from SAFS, our study found that there is power to be gained by accounting for family structure in family-based association studies of an admixed population.

## 2. Materials and Methods

**Figure 1:**
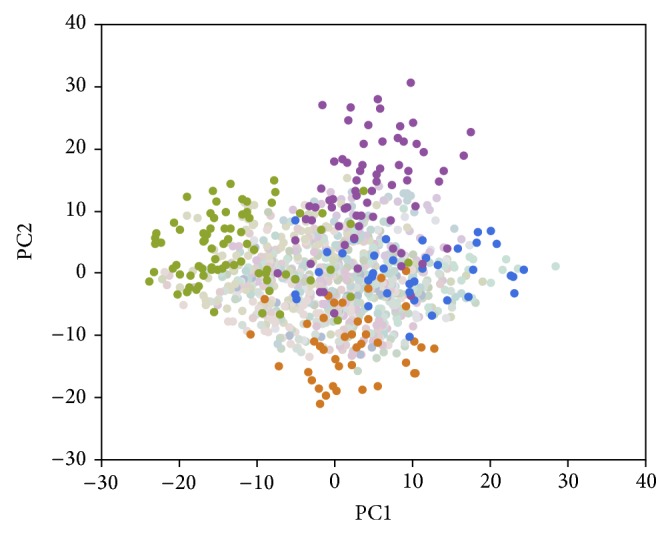
The first two principal components (PCs) using 10,000 randomly selected variants. The 20 large pedigrees from SAFS range from 22 to 86 family members.

The Genetic Analysis Workshop 18 (GAW18) data consists of whole-genome sequences from extended pedigrees [13]. The GAW18 dataset was created to provide a platform for developing and evaluating relevant statistical methods [12]. We analyzed whole-genome sequencing data from chromosome 3 and performed data cleaning to select high quality SNPs and avoid Mendelian errors. Twenty large pedigrees from SAFS ranging from 22 to 86 members in size and recruited from a Mexican American population generated 1,215,399 SNPs genotyped on 959 individuals. Mexican Americans are an admixed population with a contribution of European, Amerindian, and African ancestries [14]. We performed principal components analysis (PCA) using 10,000 randomly selected common variants (minor allele frequency (MAF) > 0.1) to investigate the family substratification. The first two principal components (PCs) revealed that families were clustered together and accounted for 2.19% and 1.47% of the total genetic variation, respectively (Figure 1). Two families (families 3 and 14 in Table 1) showed a marked difference in the first PC and two additional families (families 5 and 15) showed clear distinction in the second PC. These two pairs of families were used to select informative markers and capture the variation in this multigenerational structure pedigree. Analysis showed that 95 and 291 PCs were needed to explain 50% and 80% of the total variation, respectively. Our goal was to identify divergent family members within the extended pedigree and create a homogeneous family structure.

We calculated the MAF in each of these 4 families for the 1,215,399 SNPs. The absolute allele frequency difference (delta) was used to measure marker informativeness between the two paired divergent families mentioned above. Marker informativeness for ancestry was ascertained through the absolute value of the difference in the frequency of the minor allele from the *m*th SNP observed for the 2 populations [15]. If we let *p*_*m*1_ represent the frequency of a reference allele in the first population and *p*_*m*2_ the frequency of the same allele in the second population, then the delta value is given by *δ* = |*p*_*m*1_ − *p*_*m*2_|. Markers with different frequency distributions among populations can be used to adjust for population stratification among admixed populations. Using a cutoff value of 0.6 for delta (*δ* > 0.6, a cutoff that has been suggested as highly informative for discriminating between European and African ancestry) [16], 218 ancestry informative markers (AIMs) were selected from 1,215,399 SNPs using families 5 and 15; similarly, 404 variants were selected as AIMs for families 3 and 14. Given the vast amount of sequence data, data mining can be a fast and cost-effective approach for investigating the number of SNPs that are required to discriminate between populations. AncestrySNPminer is a web-based bioinformatics tool specifically designed to retrieve informative markers between populations with different allele frequency. The tool includes an automated and simple “scripting at the click of a button” functionality that enables researchers to perform user-friendly querying and filtering of databases across various publicly available or investigator uploaded datasets through a single web interface. The results can be downloaded or viewed in the browser where users can interactively explore linkage disequilibrium patterns and allele frequency differences among variants (https://research.cchmc.org/mershalab/) [17]. To account for population stratification, we performed PCA using all of the selected 622 AIMs to infer continuous axes of genetic variation. To assess the number of PCs needed for accurate individual family member assignment, we applied linear discriminant analysis. All analyses were run in R version 3.1.3 (http://cran.r-project.org/) [18] and packages MASS [19] (lda function) and nlme [20] (lme function) were used to run linear discriminant analysis and linear mixed modeling, respectively.

## 3. Results and Discussion

**Figure 2:**
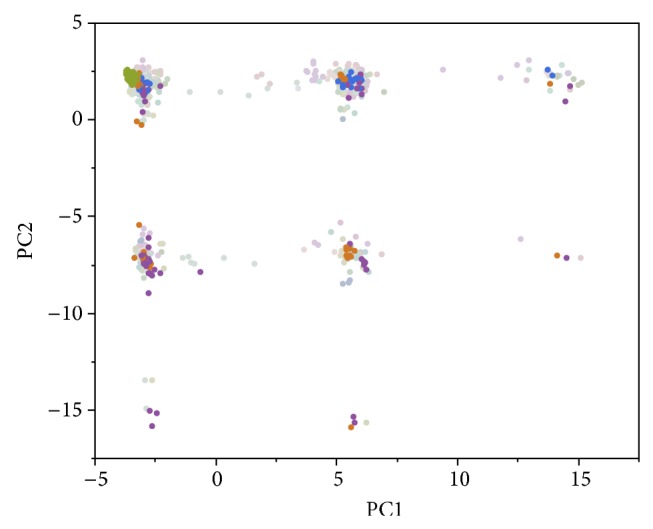
The first two principal components (PCs) using 622 AIMs. The 20 large pedigrees from SAFS range from 22 to 86 family members.

The first two principal components using the 622 AIMs accounted for 16.4% and 12.0% of the total variation, respectively, revealing an increased separation of the 20 families (Figure 2). The analysis required 8 and 35 PCs to explain 50% and 80% of the total variation, respectively. To predict family membership, we applied linear discriminant analysis on the principal components obtained from the 622 AIMs. Table 1 describes the number of family members that were correctly allocated to each corresponding family using different numbers of PCs. Notably, the number of correctly classified family members for family 3 is 77 out of 77 when using a single PC. Adding additional PCs can introduce noise but the classification accuracy levels as more PCs are utilized. Using ancestry informative markers, the classification accuracy is 12.5% for 1 PC and 92.8% for 100 PCs. In order to investigate the impact of population stratification on family-based association in an admixed population, a linear mixed model was employed to test for each SNP's association with systolic blood pressure. Blood pressure medication use, gender, age, and gender-by-age interaction were included as fixed effects in the model. We also include family as a random effect. Here, we do not explicitly model familial relationships in the random effect variance as in GRAMMAR [21] and famSKAT [22]. Based on the scree plot, we used the first six principal components (derived from 622 AIMs) as covariates to adjust for population stratification. 134 causal SNPs as simulated in GAW18 were used to assess power [13]. A null trait was used to access the Type I error based on randomly selected 10,000 SNPs.  Table 2 shows that the power was slightly improved by 1.5% after PC adjustment.  Table 2 also suggests that the Type I error was also reduced by 2.2% after we adjusted for the family substratification.

## 4. Conclusions

Accounting for population structure is more challenging when family structure or cryptic relatedness is present in an admixed population created from multiple ancestral populations. In this study, we performed population substructure analysis using extended families from admixed Mexican American population and evaluated power and Type I error in family-based association framework. Our analysis shows that power was improved by 1.5% after principal components adjustment. Type I error was also reduced by 2.2% after adjusting for family substratification. Our findings demonstrate that the traditional wisdom of family-based association studies being guarded against spurious association due to population stratification only holds when the background genetic variation is properly accounted for. The broad assumption in family-based study design is that individuals come from common genetic background among the family members. Unless this background is explicitly controlled for as in family-based association tests, or FBATs [23], robustness to population substructure may not be guaranteed. Moreover, families tend to be more homogeneous regarding exposure to environmental factors and characterized by “environmental homogeneity” that may be associated with the disease etiology. However, in an extended family of admixed population, there is also multigenerational structure created within-family and between-families, leading to unusual allele frequency differences among subgroups. Thus, it is critical to correct for stratification in family-based samples exhibiting admixture. Using an admixed population from SAFS, we accounted for relatedness and structure within admixed populations by using individual-specific allele frequencies at SNPs that are calculated on the basis of between-family variance derived from 622 SNPs serving as AIMs. Failure to appropriately account for pedigree structure can lead to spurious association (i.e., false-positive findings). Modelling family structure is a necessity in studies with family-based sample ascertainment, and there is increasing evidence that cryptic relatedness may occur in a wide range of data sets as shown in our study. Our approach offers potential solution for dealing with family structure in family-based studies. Future studies using mixed models that incorporate the full covariance structure across related individuals and model them as a polygenic component will be essential. In addition, families of Mexican American admixed genetic structure present unique opportunities to explore the genetic etiology of complex disease using admixture mapping. Mapping susceptibility genes in an admixed population using admixture mapping involves screening the genome of individuals of mixed ancestry, who have the disease, for chromosomal regions that have a greater percentage of alleles from the parental population with the higher disease risk [24]. GWAS has successfully identified common SNPs associated with many diseases. Family-based designs which include families enriched with rare genetic susceptibilities can have more power to detect genetic effects than unrelated samples given an equivalent number of sampling units [25]. Future studies integrating family-based linkage, association, and admixture mapping could help to efficiently map genomic regions associated with disease risk [26].

## Figures and Tables

**Table 1:** Number of correctly classified family members per family using different numbers of PCs. These are **boldfaced** when stably reaching the total family size.

Family ID	Number of family members	Number of PCs										
		1	20	40	60	80	100	120	140	160	180	200
2	86	10	47	67	72	75	78	81	84	**86**	**86**	**86**
3	77	77	73	76	75	76	76	**77**	**77**	**77**	**77**	**77**
4	64	0	42	49	54	57	58	61	62	62	62	63
5	68	0	54	65	65	66	66	**68**	**68**	**68**	**68**	**68**
6	64	28	33	48	50	54	56	57	58	61	62	62
7	36	0	14	19	25	26	30	33	33	33	33	33
8	68	0	37	56	60	65	66	67	67	**68**	**68**	**68**
9	33	0	10	21	27	31	30	29	31	31	31	31
10	64	0	38	50	55	59	61	61	63	63	63	**64**
11	35	0	22	25	29	30	31	33	33	34	35	34
14	40	0	25	32	33	38	39	40	39	39	**40**	**40**
15	41	0	28	33	37	38	38	39	40	40	**41**	**41**
16	48	0	31	40	41	43	42	45	45	47	46	47
17	42	5	31	33	38	37	38	39	40	42	41	**42**
20	36	0	15	24	32	31	32	32	32	36	35	35
21	35	0	20	21	29	32	33	33	33	33	34	**35**
23	32	0	15	25	27	26	29	28	31	31	30	31
25	33	0	21	25	26	27	30	30	30	30	30	31
27	35	0	18	26	27	29	33	34	34	33	34	**35**
47	22	0	19	17	19	19	19	19	21	21	21	21

**Table 2:** Improved power and reduced Type I error.

	Without PC	With PC
Power	0.142	0.157
Type I error	0.120	0.098
